# The diet-body offset in human nitrogen isotopic values: A controlled dietary study

**DOI:** 10.1002/ajpa.22140

**Published:** 2012-11

**Authors:** TC O'Connell, CJ Kneale, N Tasevska, GGC Kuhnle

**Affiliations:** 1 Department of Archaeology and Anthropology, University of CambridgeUK; 2 McDonald Institute for Archaeological Research, University of CambridgeUK; 3 MRC Dunn Human Nutrition Unit, Wellcome Trust/MRC, BuildingCambridge, UK; 4 Department of Food and Nutritional Sciences, University of ReadingUK; 5 Department of Public Health and Primary Care, MRC Centre for Nutritional Epidemiology in Cancer Prevention and Survival, University of CambridgeUK

**Keywords:** collagen, keratin, blood, nutritional biomarker, trophic level, discrimination factor

## Abstract

The "trophic level enrichment" between diet and body results in an overall increase in nitrogen isotopic values as the food chain is ascended. Quantifying the diet–body Δ^15^N spacing has proved difficult, particularly for humans. The value is usually assumed to be +3–5‰ in the archaeological literature. We report here the first (to our knowledge) data from humans on isotopically known diets, comparing dietary intake and a body tissue sample, that of red blood cells. Samples were taken from 11 subjects on controlled diets for a 30-day period, where the controlled diets were designed to match each individual's habitual diet, thus reducing problems with short-term changes in diet causing isotopic changes in the body pool. The Δ^15^N_diet-RBC_ was measured as +3.5‰. Using measured offsets from other studies, we estimate the human Δ^15^N_diet-keratin_ as +5.0–5.3‰, which is in good agreement with values derived from the two other studies using individual diet records. We also estimate a value for Δ^15^N_diet-collagen_ of ≍6‰, again in combination with measured offsets from other studies. This value is larger than usually assumed in palaeodietary studies, which suggests that the proportion of animal protein in prehistoric human diet may have often been overestimated in isotopic studies of palaeodiet. Am J Phys Anthropol, 2012. © 2012 Wiley Periodicals, Inc.

Light element isotopic analyses of human and animal body tissues are increasingly used to elucidate dietary patterns in past and living populations, with applications in archaeology, ecology, and nutritional epidemiology. However, the full potential of those analyses remains constrained by our limited understanding of the mechanisms involved in the transfer of the isotopic signature to the body during the absorption and incorporation of food. This is particularly the case with nitrogen isotopes, where there is an observed enrichment between diet and body (the "trophic level effect" or Δ^15^N_diet-body_), resulting in an increase in δ^15^N as the food chain is ascended (DeNiro and Epstein, 1981; Minagawa and Wada, 1984; Schoeninger and DeNiro,1984). Despite its clear empirical success as a dietary indicator, we do not yet know metabolically how and where the ^15^N enrichment between diet and body occurs. Ecological studies suggest that mammals, fish, birds, reptiles, and insects all have similar enrichments (Caut et al.,2009), so it seems to be independent of the mode of nitrogen excretion, but there has been little exploration of the cause. Quantifying the enrichment has proved difficult: large-scale ecological studies suggest that the enrichment associated with each trophic level is ≍+3–4‰, while small-scale animal feeding experiments show values anywhere between +1.5 and +6‰ (see review in Caut et al.,2009). In addition to being poorly quantified and understood, the trophic level effect also seems capable of quite large variation under a range of environmental conditions (temperature, altitude, aridity), as well as being potentially affected by physiological factors such as water stress, starvation and growth, digestive physiology and diet composition (for a review see McCue and Pollock, 2008).

For isotopic studies of human diet, the resolution of our interpretations is limited because we do not know what value to use for the ^15^N enrichment in humans (see Hedges and Reynard, 2007). While broad-scale changes in diet are easily observed in human isotopic values (Vogel and van der Merwe, 1977; Tauber, 1981; Buikstra and Milner, 1991; Lubell et al., 1994; Bonsall et al., 1997; Richards et al., 2003), our lack of knowledge of the Δ^15^N_diet-body_ value, and of influencing factors on this parameter, means that we cannot with confidence identify isotopic shifts resulting from small-scale dietary changes. For this, we need to quantify better the Δ^15^N_diet-body_ in humans.

## QUANTIFYING THE ENRICHMENT

It has been generally assumed that the nitrogen isotopic enrichment in mammals, including humans, is broadly similar, with a Δ^15^N_diet-body_ value initially taken to be about 3‰ (DeNiro and Epstein, 1981; Minagawa and Wada, 1984; Schoeninger and DeNiro, 1984; Hare et al., 1991), but more recently values of up to 5‰ have been postulated (Ambrose, 2000; Jenkins and Partridge, 2001; Bocherens and Drucker, 2003; Sponheimer et al., 2003; Robbins et al., 2005; Caut et al., 2009). General reviews of the ecological literature for animals ranging from invertebrates to large mammals and aquatic and terrestrial species give overall mean Δ^15^N_diet-body_ values of 2.5–3.5‰, with a high degree of variability, based on analyses of a range of body tissues (Post, 2002; McCutchan et al., 2003; Vanderklift and Ponsard, 2003). A value of around 3‰ fits with numerous predator–prey relationships in terrestrial ecological situations (see a summary in Bocherens and Drucker, 2003).

A large number of controlled animal feeding studies have been carried out, to attempt to quantify the offset (see summary in Caut et al., 2009). But for humans, the situation is more complicated, as there are significant difficulties in obtaining reliable data on which to base an estimate of human Δ^15^N_diet-body_. A number of human studies have looked at isotopic variation within populations depending on self-reported diet type (O'Connell and Hedges, 1999a; Bol and Pflieger, 2002; Petzke et al., 2005b), or compared human isotopic variation to estimated diets, either at a population level (Minagawa et al., 1986; Schoeller et al., 1986; Minagawa, 1992; Thompson et al., 2011; Valenzuela et al., 2011) or on household basis (Yoshinaga et al., 1996). A few studies have compared individuals' isotopic values to self-reported dietary records (Petzke et al., 2005a; Hedges etal., 2009; Huelsemann et al., 2009; O'Brien et al., 2009; Nash et al., 2012). Most studies of humans have used hair keratin, and some have used blood proteins (RBC, plasma, serum). Some short term feeding studies have measured other samples (such as urine and feces: Kuhnle et al., in press).

A significant problem with controlled diet isotopic studies is that of tissue turnover rates. When measuring the Δ^15^N_diet-body_, the tissues usually of interest (e.g., bone collagen, hair keratin, blood proteins) isotopically reflect medium or long-term diet (months or years), so that a short-term dietary intervention study is not possible, due to issues with tissue turnover and isotopic equilibration (Jones et al., 1981; Tieszen et al., 1983; O'Connell and Hedges, 1999a; Ayliffe et al., 2004; Huelsemann et al., 2009; Petzke and Lemke, 2009). This has long been recognized, and all robust published controlled animal feeding studies are of animals raised on a single diet over a long time period of several years, if not a lifetime. Such a study is not ethically or practically possible in humans.

Here we report isotopic analyses from humans on known and controlled diets for a short period, where the controlled diets were designed to match each individual's habitual diet, thus reducing problems with short-term changes in diet causing isotopic changes in the body pool. We measured dietary intake and a body tissue sample, red blood cells (RBCs).

## MATERIALS AND METHODS

Samples were collected from healthy subjects taking part in a 30-day dietary intervention study to develop dietary biomarkers during the period of October 2002 to June 2003. Participants were provided with their habitual diet under controlled conditions for 30 days; blood samples and duplicate diets were collected. Details of the study protocol can be found in Tasevska et al., (2005, 2006). The study was approved by the Cambridgeshire Local Research Ethics Committee (LREC No 02/232) and all participants gave their full informed written consent. Samples were archived in a controlled storage facility (Fisher Bioservice, Bishop's Stortford, UK) at −80°C for RBC and −20°C for all other specimens, and analyzed for this study in 2009–2010.

### Subjects

A total of 13 healthy subjects from Cambridgeshire, UK, were recruited with advertisements. All participants were medically examined before the beginning of the study, including an assessment of the individual's past and family medical history, details of recent and current medications, vitamin supplements, and tobacco/alcohol intake, and a cardiovascular examination. Blood analysis of fasting plasma glucose and glycated hemoglobin (HbA_1c_) was undertaken and all subjects were within the normal range (fasting plasma glucose <6.1 mmol/l, HbA_1c_ < 6%). For this study, only samples from 11 participants (five males and six females, aged 23–66 y (39.7 ± 14.7 y), with a mean BMI of 25.8 ± 4.6 kg/m^2^; Table 1) were suitable, as the 30-day study period for the remaining two was not continuous (a brief break for Christmas).

**Table 1:** Subject details, and blood and diet isotopic results

Subject	Sex	BMI	Age (y)	Mean energy intake (MJ/d)	Energy intake %CV	Mean protein intake (g/d)	Protein intake %CV	Mean N intake (g/d)	N intake %CV	Arith mean diet δ^15^N (‰)	Weighted mean diet δ^15^N (‰)	Std dev mean diet δ^15^N (‰)	Median diet δ^15^N (‰)	IQR diet δ^15^N (‰)	Blood1 δ^15^N (‰)	Blood2 δ^15^N (‰)	Mean blood δ^15^N (‰)
V1	M	27.9	52	10.9	15.2	99.1	15.4	15.4	18.2	5.5	5.5	1.2	5.2	4.8–5.8	8.9	8.8	8.9
V2	M	27.3	46	11.2	19.5	118.2	23.1	18.8	27.3	4.5	4.6	1.0	4.8	3.8–5.2	7.6	7.4	7.5
V5	F	27.5	23	10.3	16.1	82.4	31.9	12.8	21.3	4.9	5.0	0.8	4.7	4.4–5.2	8.5	8.3	8.4
V6	F	19.3	24	10.2	17.5	78.8	30	15.2	34.9	5.1	5.3	1.1	4.7	4.5–5.4	7.8	7.9	7.8
V7	M	23.1	66	15.6	19	110	29.9	18.4	33.1	4.4	4.4	1.0	4.5	3.6–5.2	8.1	8.0	8.0
V8	F	22.3	29	11.7	17.3	99	23.7	15.2	20.8	5.0	5.0	1.4	4.4	4.0–5.8	8.2	8.1	8.2
V9	M	24.5	26	11.6	25.2	110.6	42.2	20.2	37.7	4.4	4.5	1.1	4.3	3.5–5.4	7.8	7.8	7.8
V10	F	34.5	48	12.7	17.4	103.4	23	15.6	24.8	4.4	4.5	1.0	4.4	3.8–4.8	8.9	8.8	8.9
V11	M	32.2	38	14.1	21.7	120.5	24.4	18.1	25.3	4.9	4.9	1.2	4.7	4.1–5.1	8.6	8.5	8.6
V12	F	23.2	56	11.6	19.2	107.3	23.7	15.7	22.4	4.7	4.7	0.7	4.7	4.3–5.1		8.1	8.1
V13	F	22.2	29	9.4	15.9	84.8	23	12.8	21.4	5.4	5.4	1.3	5.2	4.5–5.8	8.1	8.1	8.1
Mean		25.8	39.7	11.8	18.5	101.3	26.4	16.2	26.1	4.8	4.9	1.1			8.3	8.2	8.2
Std dev		4.6	14.7	1.8	2.9	14.2	6.9	2.4	6.4	0.4	0.4	0.2			0.5	0.4	0.4
Median		24.5	38.0	11.6	17.5	103.4	23.7	15.6	24.8	4.9	4.9	1.1	4.7		8.2	8.1	8.1
IQR		22.7–27.7	27.5–50.0	10.6–12.2	16.7–19.4	91.9–110.3	23.1–30.0	15.2–18.3	21.3–30.2	4.5–5.1	4.5–5.1		4.4–4.8		7.9–8.6	7.9–8.4	7.9–8.5

### Study design

For the duration of the study, participants lived in the volunteer suite of the MRC Dunn Human Nutrition Unit (Cambridge, UK), where all food provided was prepared by trained technicians, and all specimens collected and processed. Participants followed their normal daily routine but were only allowed to consume foods prepared by the diet technicians. Subjects weighed themselves daily on an electric balance without shoes and in light clothing and recorded their body weight in the study diary. Physical activity was assessed using a questionnaire validated by the EPIC study (Wareham et al., 2003). Physical activity was recorded in the study diary on a daily basis as time (minutes) engaged in different type of exercise. A four-level score (inactive, moderately inactive, moderately active, and active) was assigned by combining occupational physical activity together with time participating in higher-intensity physical activities such as cycling, aerobics, swimming, jogging, exercising at a gym on a regular basis, etc.

### Diets

Prior to the study, participants were asked to keep 7-day food diaries for 4 weeks while living at home. Weekly interviews with one of the investigators provided additional information, such as brand names. These data were used to replicate the habitual diet of each participant for the duration of the study. From approximately two-and-a-half times the amount of food expected to be eaten by the participant, one-half was prepared and one-half was kept for the preparation of a duplicate meal. The prepared half was weighed to the nearest gram, labeled with the name and the day, and left in a separate refrigerator for each individual. During the day, participants helped themselves and returned the uneaten food to the containers in the refrigerator. The next day, the uneaten food was weighed out and the amount of food eaten was calculated.

Dietary intake was calculated from the UK food-composition tables using DINER (Data Into Nutrients for Epidemiological Research) (Welch et al., 2001). Tea and coffee were consumed freely during the course of the study, but participants were asked to keep their intake consistent and estimated intake was included in the data analysis. Five of the participants occasionally consumed alcohol; as this was not permitted in the volunteer suite, participants consumed alcohol outside the premises and recorded amount and type. The calculated dietary intake for alcoholic drinks was also added into the consumption data obtained in the study.

Duplicate diets were prepared daily for each participant. All food and drink items (excluding coffee, tea, alcoholic drinks, water, added salt, and pepper) were weighed to the nearest 1 g, chopped up and crushed, mixed with a weighed amount of boiling deionized water, and homogenized with a Magimix 5100 automatic food processor, usually for 10–15 min, until a smooth emulsion was obtained. Aliquots of each duplicate were stored at –20°C for analysis.

### Blood collection, handling, and storage

Blood was sampled twice from each subject, at the start and in the last week of the study, by a trained phlebotomist. For one subject (V12), only blood collected at the end of the study was available for analysis. Fasting venous blood was collected into 10 ml lithium heparin monovettes. Within 1 h, the monovettes were centrifuged, the red blood cells removed from below the LiHep beads, washed thrice in chilled physiological solution, and then stored at −80°C prior to analysis.

### Isotopic analyses

Duplicate diet samples were analyzed as liquid homogenates representative of 24 h food intake for each individual's diet. Eight to twelve days' diets were analyzed per subject, from the last half of the study. Samples were lyophilized and weighed into tin capsules (0.8 mg per aliquot). Red blood cell samples (0.2 ml) were lyophilized and then weighed into tin capsules (0.8 mg per aliquot). Diet samples were isotopically analyzed in duplicate, while blood samples were run in triplicate.

Isotopic analyses were performed using a Costech (Valencia, CA) automated elemental analyzer coupled in continuous-flow mode to a Thermo Finnigan MAT253 (Bremen, Germany) mass spectrometer at the Godwin Laboratory, Department of Earth Sciences, University of Cambridge. Stable isotope concentrations are measured as the ratio of the heavier isotope to the lighter isotope relative to an internationally defined standard, AIR (Hoefs, 1997). Isotopic results are reported as δ^15^N values in parts per 1000 or "permil" (‰) values, where δ^15^N = [(^15^N/^14^N _sample_/^15^N/^14^N _standard_) − 1] × 1,000. Based on replicate analyses of international and laboratory standards, measurement errors are less than ±0.2‰ for δ^15^N.

### Statistical analysis

Because of the sample size and distribution of the data, nonparametric tests were conducted to investigate differences. The main objective of this study was to investigate differences in δ^15^N between diet and blood; assuming a standard deviation of 10% (higher than observed in this study) and a sample size of 11, changes of 15% can be detected with a power (1-β) of 0.9 at a significance level of α = 0.05. Power calculations were performed with G*Power 3.1.2 (Faul et al., 2009). Data analyses were conducted using Stata 11.2 (Statacorp, College Station, TX). The bivariate boxplot (bagplot: Rousseeuw etal., 1999) was prepared in R 2.12.1 (Team, 2009). Unless indicated otherwise, data are given as mean ± standard deviation.

## RESULTS

Results are shown in Table 1. Overall, the body weight remained constant throughout the study (75.6 ± 15.7 kg at start vs*.* 75.8 ± 15.6 kg at end; Wilcoxon signed rank test, *P* = 0.56) which suggests that the intake achieved in the study was a valid reflection of the usual dietary habits in these volunteers. Weight changed by less than 2% in 10 participants; in one participant, the weight increased from 63.1 kg to 64.8 kg. However, this can be explained by normal fluctuations in the body weight, and changes in activity patterns during the study. Thus we take this population as being in a good approximation to steady state. True steady-state conditions are rarely achieved in free-living individuals, because abrupt changes in nitrogen balance occur from day to day, related to changes in dietary intake. Net accumulations and loss in nitrogen can be as much as ±2SE for free-living individuals, largely due to day-to-day variations in dietary nitrogen intake which can take several days to be reflected in excreted nitrogen (Bingham and Cummings, 1985). Of the 11 subjects, three of the subjects were physically inactive, three moderately inactive, four moderately active, and one active. They mostly practiced cycling, swimming, exercising at the gym, and jogging.

The median diet nitrogen isotopic value for all subjects was 4.7‰ (range in subject medians of 4.3–5.2‰). The mean diet nitrogen isotopic value for all subjects was 4.8 ± 0.4‰ (range in subject means of 4.4–5.5‰). We investigated whether daily variation in dietary nitrogen content would affect the average dietary nitrogen isotopic value for each subject, since individuals did not consume the same amount of protein on each of the 30 days of the study. For nine of the subjects, the difference between the arithmetical mean δ^15^N and the mean δ^15^N of each subject's diets weighted by the nitrogen contribution from each day's diet was less than 0.1‰, and for two individuals, the difference was less than 0.2‰; overall there was no statistically significant difference (Wilcoxon signed rank test, *P* = 0.37) between the two means (Table 1), so we consider that varying nitrogen intake had little if any quantifiable effect. Total protein intake and total nitrogen intake were inversely correlated with diet δ^15^N, although this correlation was only marginally significant (Spearman rank correlation: ρ= −0.59, *P* = 0.05, and ρ = 0.57, *P* = 0.07, respectively).

The range of RBC nitrogen isotopic values for all subjects was 7.6–8.9‰ at the start of the study and 7.4–8.8‰ at the end of the study. The median δ^15^N_RBC_ for all subjects was 8.2‰ (IQR= 7.9–8.6‰) at the start of the study, and 8.1‰ (IQR = 8.0–8.4‰) at the end of the study; the mean δ^15^N_RBC_ for all subjects was 8.3 ± 0.5‰ at the start of the study, 8.2 ± 0.4‰ at the end of the study, and 8.2 ± 0.4‰ for the two values averaged. Comparison of the δ^15^N_RBC_ of blood taken at the start and end of the study shows a small decrease (comparison possible for 10 of the 11 subjects: median difference = −0.1‰, Wilcoxon test, *P* = 0.02; Table 1).

**Fig 1:**
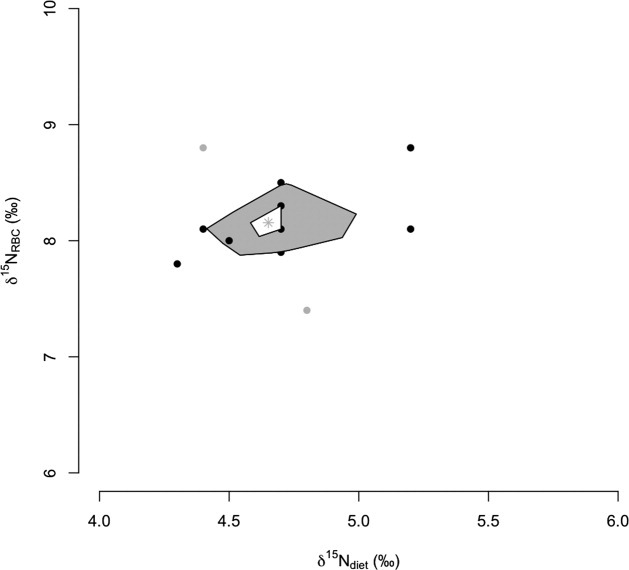
Nitrogen isotopic values of red blood cells and study diets for each subject. Data for the final blood sample and the median diet for each individual are shown as a bagplot (Rousseeuw et al., 1999): 50% of samples are within the gray area, the median is marked as a star, the central white region is a 95% confidence region for the depth median of the group, and the two identified outliers are shown in gray.

The overall difference between blood RBC and diet δ^15^N (Δ^15^N_diet-RBC_) in the population can be calculated in several ways, depending on whether the mean or median for the population is used (Table 2). The range of individual Δ^15^N_diet-RBC_ is between 2.7 and 4.4‰, whichever way is used, and the average Δ^15^N_diet-RBC_ for the group is between +3.3 and +3.6‰, with the statistically most parsimonious value (using the final blood sample δ^15^N_RBC_ and the median diet δ^15^N) of +3.5‰ (Fig. .1). We did not observe any statistically significant difference between men and women, and no significant correlation with age or physical activity. The study was carried out over a period of months, but the sample size was too small to investigate the possible effects of seasonal changes in metabolic activity. However, Δ^15^N_diet-RBC_ and δ^15^N_RBC_—but not δ^15^N_diet_—correlated significantly with BMI (Spearman rank correlations, respectively: ρ = 0.73, *P* = 0.02; ρ = 0.62, *P* = 0.04; ρ = −0.29, *P* = 0.38). If we exclude those who are obese (BMI > 30), all three correlations are non-significant, thus it is possible that the two obese subjects skew the data. For the nine subjects with BMI < 30 (nonobese) the mean Δ^15^N_diet-RBC_ is +3.2‰, as compared with +3.4‰ for all 11 subjects, using the mean δ^15^N values of diet and RBC.

**Table 2:** The Δ^15^N_diet-RBC_ of the population calculated in different ways, using the mean and median measures of the subjects' nitrogen isotopic values

Subject	Arith mean δ^15^N_diet_ (‰)	Median δ^15^N_diet_ (‰)	Blood 2 δ^15^N_RBC_ (‰)	Mean δ^15^N_RBC_ (‰)	Δ^15^N_diet-RBC_ (mean blood - mean diet) (‰)	Δ^15^N_diet-RBC_ (blood 2 - mean diet) (‰)	Δ^15^N_diet-RBC_ (mean blood - median diet) (‰)	Δ^15^N_diet-RBC_ (blood 2 - median diet) (‰)
V1	5.5	5.2	8.8	8.9	3.3	3.3	3.7	3.6
V2	4.5	4.8	7.4	7.5	3.0	2.9	2.7	2.7
V5	4.9	4.7	8.3	8.4	3.4	3.3	3.7	3.6
V6	5.1	4.7	7.9	7.8	2.7	2.7	3.1	3.1
V7	4.4	4.5	8.0	8.0	3.7	3.6	3.6	3.5
V8	5.0	4.4	8.1	8.2	3.2	3.1	3.7	3.7
V9	4.4	4.3	7.8	7.8	3.5	3.5	3.5	3.5
V10	4.4	4.4	8.8	8.9	4.4	4.4	4.4	4.4
V11	4.9	4.7	8.5	8.6	3.7	3.7	3.9	3.8
V12	4.7	4.7	8.1	8.1	3.4	3.4	3.4	3.4
V13	5.4	5.2	8.1	8.1	2.8	2.8	2.9	2.9
Mean	4.8		8.2	8.2	3.4	3.3	3.5	3.5
Stdev	0.4		0.4	0.4	0.5	0.5	0.5	0.4
Median	4.9	4.7	8.1	8.1	3.4	3.3	3.6	3.5
IQR	4.5–5.1	4.4–4.8	7.9–8.4	7.9–8.5	3.1–3.6	3.0–3.5	3.2–3.7	3.3–3.7
Max	5.5	5.2	8.8	8.9	4.4	4.4	4.4	4.4
Min	4.4	4.3	7.4	7.5	2.7	2.7	2.7	2.7

## DISCUSSION

The assumption underlying the premise of this study is that the controlled diet consumed by subjects over the 30-day study was isotopically similar to their habitual diets. The study for which these samples were collected was not designed as an isotopic study, so no consideration was made of isotopic variability in foods. However, the diets were carefully designed so as to match the composition of habitual diets, including the matching of brands consumed. A small but significant average decrease of 0.1‰ in δ^15^N_RBC_ suggests that the study diets were not isotopically identical to habitual diets (bearing in mind that each subject's study diet was specific to them, so some may have been different and others not). Red blood cells have a mean in vivo life span of 120 days (Landaw, 1991), so a median change of −0.1‰ in δ^15^N_RBC_ over the duration of the 30-day study suggests that there could be a median difference of −0.4‰ over 120 days. Thus the measured δ^15^N_RBC_ of bloods taken at the end of the study may be an overestimate by +0.3‰ compared with that which would be measured if the subjects continued on the controlled diets for several months. Therefore we suggest that the range of Δ^15^N_diet-RBC_ values that we derive, of +3.3 to +3.6‰ (Table 2), should be expanded to be +3.0–3.6‰, but that Δ^15^N_diet-RBC_ is highly likely to be larger than +3‰. For the further discussion in this paper, we use the value of +3.5‰, based on the most parsimonious value of Δ^15^N_diet-RBC_, with the recognition that it may be a slight overestimate.

Studies have shown that isotopic differences between diet and animal tissues can vary under different conditions (e.g., Ambrose and DeNiro, 1986; Heaton et al., 1986; Sealy et al., 1987; Hobson and Clark, 1992; Hobson et al., 1993; Gröcke et al., 1997), and that human nitrogen isotopic values vary under different conditions, including pregnancy, growth, illness and pathology (e.g., Katzenberg and Lovell, 1999; Fuller et al., 2004; Fuller et al., 2005; Mekota et al., 2006; Waters-Rist and Katzenberg, 2010). Thus it is likely that the offset measured here will not be universally constant for all humans on all diets. However, this is the first quantified isotopic study of the diet to body enrichment in humans on controlled diets, and therefore gives an indication of the magnitude of the offset that we can expect. We found no effect of sex or age on Δ^15^N_diet-body_ offset in these subjects. The observed positive correlation with BMI, driven by the two obese subjects, is intriguing and requires further investigation: the possibility of an effect of differential bioavailability of nutrients and differential uptake between individuals may be a factor here, and one that should be considered further.

### Offsets from diet to keratin and collagen

To be able to use this measured diet-body offset for humans in palaeodietary studies, we must estimate what it equates to in terms of tissues analyzed in other studies, such as keratin or collagen. We can combine our data with that of three other studies, all on North American residents, to derive a value for Δ^15^N_diet-keratin_ (Table 3). Nash et al. (2009) showed a mean increase of +1.5 ± 0.6‰ from RBCs to hair keratin. Kraft et al. (2008) showed that blood plasma has a higher δ^15^N than red blood cells by 1.5‰ on average. Schoeller et al. ( 1986) showed a mean increase of +0.3 ± 0.7‰ from plasma protein to hair keratin. Combining the plasma/RBC/keratin results from these two latter studies, we get an estimated offset of +1.8‰ from RBCs to hair keratin, in fairly good agreement with the value of +1.5‰ observed by Nash et al. Our measured Δ^15^N_diet-RBC_ value of +3.5‰ equates to a Δ^15^N_diet-keratin_ of ≍+5.0‰ using the Nash offset, and to ≍+5.3‰ using the Kraft-Schoeller combined offset (no errors propagated).

**Table 3:** Nitrogen isotopic values of tissues, diet and calculated diet-tissue offsets in published human studies (all given in units of ‰)

	Yoshinaga et al., 1996					Hedges et al., 2009	Nash et al., 2009	Kraft et al., 2008	Schoeller et al., 1996	O'Connell et al., 2001	O'Connell and Hedges, 1999	Richards, 2006
Population	Rual, PNG	Wonie, PNG	Ume, PNG	Dorogi, PNG	Fiji	Alaska	Baltimore	Chicago	Oxford	UK (archaeol)	UK (archaeol)
Sex	M	M	M	M	F	M&F	nd	M&F	M&F	M&F	M&F
N	15	13	10	11		144	31	9	8	23	13
Hair δ^15^N	9.1 ±0.5	8.9 ±0.7	9.4 ±0.6	11.3 ± 0.6	8.8 ± 0.3	10.8 ± 1.9		9.7 ±0.5 a	9.5 ± 0.7	10.6 ± 1.4	10.9 ± 1.3
Bone collagen δ^15^N									10.3 ± 0.6	11.6 ± 1.7	12.0 ± 0.8
RBC δ^15^N						9.3 ± 1.7	7.2^b^				
Plasma δ^15^N							8.7^b^	9.4 ± 0.5^a^			
Diet δ^15^N	3.3	2.0	4.3	6.3	4.7 ± 0.3						
Δ^15^N_diet-keratin_	+5.8	+6.9	+5.1	+5.0	+4.1 ± 0.7						
Δ^15^N_RBC-keratin_						+1.5 ± 0.6					
Δ^15^N_RBC-plasma_							+1.5^b^				
Δ^15^N_plasma-keratin_								+0.3 ± 0.7^a^			
Δ^15^N_keratin-collagen_									+0.9 ± 0.2	+1.0 ± 1.1	+1.0 ± 1.4

a Mean values calculated from the individual subject data, rather than the reported averages in Table 4 of the paper.

b Values taken from Table 3b of the paper, where the mean but no standard deviations are given.

Our derived Δ^15^N_diet-keratin_ value can be compared to estimates from two studies specifically examining the offset from diet to hair keratin, based on estimates of dietary intake combined with food and hair isotopic analysis (Table 3). Yoshinaga et al. ( 1996) analyzed 49 males in Papua New Guinea, in the period 1980–1982. Through food consumption surveys, food isotopic analysis, and hair isotopic analysis, they derived an estimated value of +5.0–6.9‰ for Δ^15^N_diet-keratin_ based on a calculated diet for each individual. Hedges et al. ( 2009) analyzed 20 females in Fiji sampled in 1999. Through diet diaries, food isotopic analysis, and hair isotopic analysis, they derived an estimated value of +4.1 ± 0.7‰ for Δ^15^N_diet-keratin_ based on a calculated diet for each individual. Our measured data with a combination of the Nash-Jahren-Schoeller offsets gives an estimate of Δ^15^N_diet-keratin_ of +5.0–5.3‰, which falls between the estimated values from Yoshinaga and Hedges. Studies estimating dietary intake at the population level have estimated a Δ^15^N_diet-keratin_ of ca. +4.3‰ (Minagawa et al., 1986; Schoeller et al., 1986).

To consider how our data would translate to a Δ^15^N_diet-collagen_ offset, we must then consider the offset between human hair keratin and bone collagen. Three published studies have measured this in humans, one in a modern population (+0.9 ± 0.2‰: O'Connell etal., 2001) and two in archaeological individuals (+1.0 ± 1.1‰: O'Connell and Hedges, 1999b); +1.0 ± 1.4‰: (Richards, 2006) (Table 3). There are problems in using such data (such as the small sample sizes and the large standard deviations in the two archaeological studies) but it is noteworthy that all studies have similar mean offsets for the Δ^15^N_keratin-collagen_ offset. Adding these corrections to the estimated Δ^15^N_diet-keratin_ of +5.0–5.3‰ derived from our data and the offsets measured by Nash/Kraft/Schoeller et al., we derive a range of +5.9–6.3‰ for the Δ^15^N_diet-collagen_ offset (again no errors propagated).

As we discuss earlier, the measured δ^15^N_RBC_ may be an overestimate, and thus the derived values of Δ^15^N_diet-keratin_ and Δ^15^N_diet-collagen_ may also be overestimated. Possible problems with studies comparing keratin to diet include issues with growth cycle errors (Williams et al., 2011). Problems with studies comparing collagen and keratin include differential time periods represented in the two tissues (O'Connell et al., 2001; Hedges et al., 2007). However, even with a very conservative approach, assuming a Δ^15^N_diet-RBC_ value of +3‰, and using minimum offset values to keratin (Nash study, +0.9‰, i.e., 1σ less than the mean), and to collagen (O'Connell 2001 modern study, +0.7‰, i.e., 1σ less than the mean), our results suggest a Δ^15^N_diet-collagen_ offset of +4.6‰, which is at the upper end of the currently accepted range. These data suggest therefore a larger offset than commonly assumed.

We can place the limited human data in the context of that from other animal studies. All controlled feeding studies on animals so far have observed isotopic inhomogeneity in different tissues, and such isotopic differences can be substantial (Caut et al., 2009). Other mammalian studies have shown a similar pattern to that summarized above for humans: whole blood and red blood cells generally have low nitrogen isotopic values relative to other tissues, or at the low end of the range, and in comparisons of plasma and red blood cells, plasma always has a higher nitrogen isotopic value, often by more than 1‰ (Table 4). As regards the magnitude of the offsets, similar values to our estimates are found for a range of species in the literature. A number of animal studies have found Δ^15^N_diet-body_ differences of greater than 4‰ for a variety of tissues (DeNiro and Epstein, 1981; Hilderbrand et al., 1996; Roth and Hobson, 2000; Sponheimer et al., 2003; Arneson and MacAvoy, 2005; Miron et al., 2006; Caut etal., 2008), and studies of goat, alpaca, seal and bear have shown differences larger than 5‰, up to 6.4‰ (Kurle, 2002; Felicetti et al., 2003; Sponheimer etal., 2003).

**Table 4:** Nitrogen isotopic offsets between diet, blood and other tissues in published controlled mammal feeding studies

Reference	Species common name	Δ^15^N_diet-tissue_ (‰)							
		Liver	Muscle	Hair	Whole Blood	RBC	Plasma
Nakagawa et al., 1985	Rats	2.6		1.6	1.5		
Arneson et al., 2005	Mice	4.3	3.1		3.2		
Arneson et al., 2005	Mice	4.7	3.1		3.2		
Arneson et al., 2005	Mice	3.8	2.0		2.9		
Hobson et al., 1996	Harp, Harbor, Ringed seals	3.1	2.4	3.0		1.7	
Kurle, 2002^a^	Northern fur seals					3.9	5.2
Lesage et al., 2002	Gray, Harbor, Harp seals					1.7	3.1^b^
Lesage et al., 2002	Harp seals					2.0	3.6
Roth and Hobson, 2000	Red fox	3.6	3.6	3.4		2.6	4.2
Yoneyama et al., 1983	Rats	3.4	3.1			2.0	3.9

a Data from 5 seals, excluding that from pregnant/lactating Baabs.

b Data measured on serum, not plasma.

### Implications of this study for palaeodietary work

Overall, our data suggest that the Δ^15^N_diet-collagen_ offset in this group is ca. +6‰, larger than that usually assumed in the archaeological literature, typically around +3-5‰ (Bocherens and Drucker, 2003). Using a very conservative approach to the data, the estimate is still ca. +4.6‰, at the upper end of the currently accepted range. Such an observation has implications for the interpretation of human palaeodiet from isotopic data: an underestimation of the Δ^15^N_diet-collagen_ offset will lead to an overestimation of the dietary importance of foods with higher nitrogen isotopic values, usually higher trophic level foods such as meat, milk and fish. As Hedges and Reynard ( 2007) note, using a Δ^15^N_diet-collagen_ value of 3-4‰ produces an estimate of dietary animal protein percentage (as a proportion of total protein intake) of 60% and sometimes up to 80% for prehistoric farmers in Europe, which is greater than animal protein dietary fraction of modern "developed" countries and twice that of modern "developing" countries (Sluijs et al.; Frassetto et al., 2000; FAOSTAT, 2012), as well as being in excess of that consumed by most ethnographically documented hunter-gatherer populations (Cordain et al., 2000). If a value of +6‰ were used as Δ^15^N_diet-collagen_ offset, this would typically reduce the dietary animal protein intake estimate by about a third to a half, bringing such estimates for prehistoric farmers in line with dietary animal/plant protein ratios in living horticultural/agricultural populations (Yoshinaga et al., 1996; Frassetto et al., 2000; MacIntyre etal., 2002; Muhammad-Lawal and Balogun, 2007; Hedges et al., 2009; Iyangbe and Orewa, 2009; Baroudi et al., 2010).

## CONCLUSIONS

In 11 subjects consuming their habitual diets under controlled conditions, we have measured the Δ^15^N_diet-RBC_ as +3.5‰. This is the first study to measure the Δ^15^N_diet-body_ offset in humans on controlled diets of known isotopic composition. Using measured offsets from other studies, we estimate the human Δ^15^N_diet-keratin_ as +5.0−5.3‰, which is in good agreement with estimates derived from the two other studies using individual diet records (Yoshinaga et al., 1996; Hedges et al., 2009). We also derive a value for Δ^15^N_diet-collagen_ of ≍6‰, larger than usually assumed in palaeodietary literature. This larger value goes some way to resolving the conundrum of interpretations of very high animal protein intake in isotopic studies of prehistoric farmers—we suggest that this has often been overestimated. We advocate that dietary interpretations of previously published archaeological human isotopic data are reconsidered in the light of our work.
